# Interaction of Cortical and Amygdalar Synaptic Input Modulates the Window of Opportunity for Information Processing in the Rhinal Cortices

**DOI:** 10.1523/ENEURO.0020-19.2019

**Published:** 2019-08-23

**Authors:** Janske G. P. Willems, Wytse J. Wadman, Natalie L. M. Cappaert

**Affiliations:** Center for NeuroScience, Swammerdam Institute for Life Sciences, University of Amsterdam, Amsterdam 1098 XH, The Netherlands

**Keywords:** entorhinal cortex, parvalbumin interneurons, patch clamp, perirhinal cortex, voltage-sensitive dye imaging

## Abstract

The perirhinal (PER) and lateral entorhinal (LEC) cortex function as a gateway for information transmission between (sub)cortical areas and the hippocampus. It is hypothesized that the amygdala, a key structure in emotion processing, can modulate PER-LEC neuronal activity before information enters the hippocampal memory pathway. This study determined the integration of synaptic activity evoked by simultaneous neocortical and amygdala electrical stimulation in PER-LEC deep layer principal neurons and parvalbumin (PV) interneurons in mouse brain slices. The data revealed that both deep layer PER-LEC principal neurons and PV interneurons receive synaptic input from the neocortical agranular insular cortex (AiP) and the lateral amygdala (LA). Furthermore, simultaneous stimulation of the AiP and LA never reached the firing threshold in principal neurons of the PER-LEC deep layers. PV interneurons however, mainly showed linear summation of simultaneous AiP and LA inputs and reached their firing threshold earlier. This early PV firing was reflected in the forward shift of the evoked inhibitory conductance in principal neurons, thereby creating a more precise temporal window for coincidence detection, which likely plays a crucial role in information processing.

## Significance Statement

The perirhinal (PER) and lateral entorhinal (LEC) cortices function as a gateway for information transmission between the neocortex and the hippocampus and this information flow can be modulated by the amygdala. Here, we showed that simultaneous input of the neocortex and the amygdala coincided onto principal neurons and parvalbumin (PV) interneurons of the PER-LEC deep layers. PV interneurons linearly summated these synaptic inputs and reached their firing threshold earlier. This earlier PV firing resulted in an earlier rise of the inhibitory conductance in principal neurons, likely causing a more precise temporal window for excitatory coincidence detection. This process probably indicates a significant role for the inhibitory network in regulating integration of emotion and information for processing in the PER-LEC deep layer network.

## Introduction

The parahippocampal region is a crucial part of the memory system. The subregions of this parahippocampal pathway, the perirhinal (PER) and lateral entorhinal (LEC) cortex, function as the gateway between the (sub)cortical and the hippocampal formation to ensure memory formation and retrieval (Burwell and Amaral, 1998; Burwell, 2000; Burwell and Witter, 2002; Fernández and Tendolkar, 2006). Although structural connections between the neocortex and the hippocampus through the PER and LEC exist (Cappaert et al., 2014), information transfer occurs with a low probability (Biella et al., 2002; Pelletier et al., 2005; Koganezawa et al., 2008). How the probability of information transfer can be modified remains to be revealed, but evidence suggests that the amygdala might play a role.

Emotional enhancement of memory is an important feature of the memory system and plays a crucial role in the survival of species (Christianson, 1992). It has been shown in animal as well as in human studies that the amygdala can modulate medial temporal lobe activity (including the PER and LEC) and therefore enhances memory performance on emotional versus neutral stimuli (Cahill and McGaugh, 1998; Kilpatrick and Cahill, 2003; Dolcos et al., 2004). As so, it is shown that the amygdala can modulate the rhinal gate in a way that information from the neocortex is more reliably transmitted through the PER-LEC circuitry (Paz et al., 2006).

Previous studies showed that neocortical and amygdala stimulation both lead to PER-LEC neuronal population activity and *in vivo* (Martina et al., 2001; Pelletier and Paré, 2002; Biella et al., 2003; Pelletier et al., 2005) and in brain slices under the condition of partial GABA_A_ blockade (Kajiwara et al., 2003; Koganezawa et al., 2008; Willems et al., 2016). Furthermore, amygdala-evoked activity can promote the propagation of network activity from the PER through the LEC into the dentate gyrus of the hippocampus *in vitro* (Kajiwara et al., 2003; Koganezawa et al., 2008), once the inputs from the PER and amygdala coincide in the deep layers of the LEC (Koganezawa et al., 2008). Field recordings in the PER showed that amygdala activation increases responsiveness of PER neurons to neocortical stimuli *in vivo* (Pelletier et al., 2005). Nevertheless, how the interaction of synaptic inputs of the neocortex and the amygdala on PER and LEC neurons results in increased neuronal responsiveness is not yet understood. It is hypothesized that simultaneous activation of the amygdala and neocortex can result in altered excitatory; inhibitory properties of the PER-LEC network.

A key player to modulate the neuronal excitability in the PER-LEC is the local inhibitory network (Willems et al., 2018). Especially parvalbumin (PV)-positive interneurons are candidates for efficient inhibitory control, as they project onto the soma and axon initial segment of principal neurons (Pfeffer et al., 2013). It has been shown that the amygdala can regulate principal neuron excitability in the prefrontal cortex via recruitment of PV interneurons in the local prefrontal cortex circuit, resulting in fast feedforward inhibition, which regulates emotional behavior (McGarry and Carter, 2016). The PV interneurons might have a similar function in the modulation of activity in PER-LEC deep layers. However, understanding of how these interactions are processed at a local circuit level is still lacking.

To examine the interactions of the neocortex and the amygdala in the PER-LEC, the current study stimulated the agranular insular cortex (AiP) and lateral amygdala (LA) to represent neocortical and amygdalar synaptic input, respectively. The AiP is a neocortical area involved in emotional, interoceptive and exteroceptive signal processing (Nieuwenhuys, 2012) and the amygdala plays a pivotal role in emotion processing (LeDoux, 2000). These brain areas have been shown to project to the PER-LEC network (Krettek and Price, 1977; Burwell and Amaral, 1998; Pitkänen et al., 2000; Canto et al., 2008; Kealy and Commins, 2011; Mathiasen et al., 2015) and the connections are present in the horizontal brain slice preparations used in this study (von Bohlen und Halbach and Albrecht 2002; Mathiasen et al., 2015). In this study we showed how the inhibitory and excitatory network is recruited by synaptic input originating from the AiP and LA. Furthermore, we examined how synaptic inputs, originating in the AiP and LA, interact on PER-LEC deep layer principal neurons and PV interneurons. Both neuron types received synaptic input from the LA as well as the AiP and interaction of inputs in PV interneurons triggered a forward shift in spike timing, causing the temporal window of opportunity for coincidence detection of excitation to be more precise.

## Materials and Methods

### Animals

Experiments were performed on male and female C57BL/6 mice (Harlan Laboratories) and male and female Pvalb^tm1(cre)Arbr^ (Hippenmeyer et al., 2005)/Gt(ROSA)26Sor^tm1(EYFP)Cos^ (Srinivas et al., 2001) transgenic mice. All animals were between the ages of P28 and P42. All animal procedures were performed in accordance with the University of Amsterdam, animal care committee’s regulations.

### Slice preparation

Horizontal slices (400 µm thick) containing the neocortical AiP, LA, PER, and LEC were cut using a VT1200S vibratome (Leica Biosystems). Functional projections from the AiP and the LA to the PER and EC are present in this slice preparation (von Bohlen und Halbach and Albrecht 2002; Mathiasen et al., 2015).

Whole-cell recordings: animals were killed by decapitation, whereafter the brain was rapidly removed and stored in ice-cold artificial CSF (ACSF) containing the following (in mm): 120 choline chloride, 3.5 KCl, 5 MgSO_4_, 1.25 NaH_2_PO_4_, 0.5 CaCl_2_, 25 NaHCO_3_, 10 d-glucose, pH 7.4, 300–315 mOsm, oxygenated with 95% O_2_/5% CO_2_ for at least 30 min. The slices were incubated for 15 min after sectioning at 32°C in artificial CSF containing the following (in mm): 120 NaCl, 3.5 KCl, 1.3 MgSO_4_, 1.25 NaH_2_PO_4_, 2.5 CaCl_2_, 25 NaHCO_3_, 10 d-glucose, oxygenated with 95% O_2_/5% CO_2_, pH 7.4, 300–315 mOsm. Thereafter slices were kept at room temperature until the recording started.

Voltage-sensitive dye imaging: the mice were decapitated and the brain was stored in ice-cold artificial CSF containing the following (in mm): 120 NaCl, 3.5 KCl, 5 MgSO_4_, 1.25 NaH_2_PO_4_, 2.5 CaCl_2_, 25 NaHCO_3_, and 10 d-glucose oxygenated with 95% O_2_/5% CO_2_, pH 7.4. After slicing, the brain slices were acclimatized for 30 min and subsequently slices were incubated for 1 h at room temperature with 0.007 mg/ml of the oxonol VSD, NK3630 (Hayashibara Biochemical Laboratories). After staining, the slices were kept at room temperature in a holding chamber on a membrane (Millipore LCR membrane filter, FHLC02500, Polytetrafluoroethylene hydrophilic membrane with 0.45 µm pore size, Millipore), placed on a well filled with ACSF in a moistened 95% O_2_/5% CO_2_ atmosphere.

### Electrical stimulation

**Figure 1.**
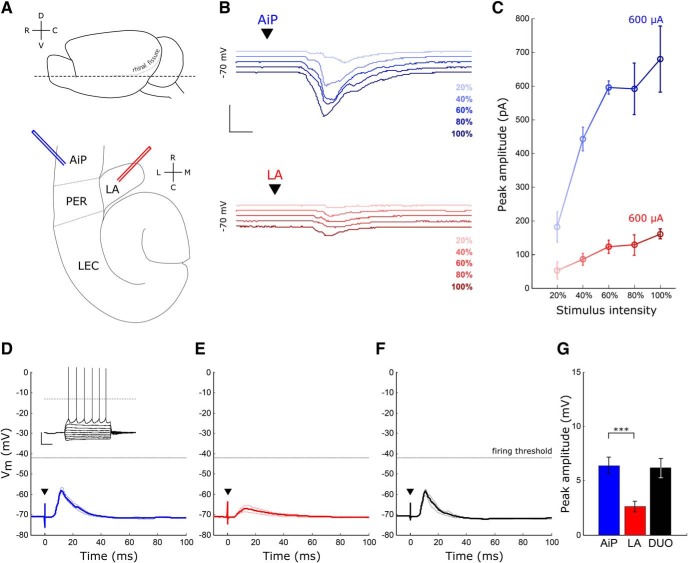
Evoked synaptic responses in principal neurons after AiP and LA stimulation. ***A***, Schematic overview of the horizontal slice preparation. Top, Lateral view of the mouse brain, the dotted line indicates the horizontal slice cut. Bottom, Overview of a horizontal slice with the placement of the AiP (blue) and LA (red) stimulus electrode. ***B***, Typical example of synaptic responses to five increasing stimulus intensities in the AiP (blue) and LA (red) in a deep layer principal neuron. Scale bars: 400 pA, 5 ms. ***C***, Example input/output curve of the synaptic currents in the neuron shown in ***B***. Error bars represent SEM over three consecutive repeats. ***D***, Typical example of an evoked postsynaptic potential after AiP stimulation in a principal neuron; inset shows the action potential firing evoked by current injection. Scale bars: 25 mV, 250 ms. ***E***, ***F***, Typical example of an evoked postsynaptic potential after LA stimulation (***E***) and DUO stimulation (***F***) in the same principal neuron as in ***D***. ***G***, The mean evoked postsynaptic potential peak amplitude of all neurons recorded (*n* = 31). Asterisks indicate the significance level (***p < 0.001). D, Dorsal; C, caudal; V, ventral; R, rostral; L, lateral; M, medial; ▼ indicates the moment the stimulus was applied.

Stimulation electrodes were placed under visual guidance in the superficial layers of the AiP and in the LA (Fig. 1*A*). For electrical stimulation in whole-cell recordings, a bipolar tungsten stimulus electrode (World Precision Instruments) with a tip separation of 125 µm was placed under visual guidance in the superficial layers of the AiP and in the LA. A single biphasic stimulus pulse (160 µs/phase) was applied using a DS4 biphasic current stimulator (Digitimer). Electrical stimuli in the voltage-sensitive dye experiments were applied through a custom-made bipolar stimulation electrode (100 μm diameter isolated stainless steel wire) with a tip separation of 150–200 µm. Biphasic square pulses of 0.3 ms were applied through a custom-made current source with amplitudes of 500 μA.

### Voltage-sensitive dye imaging

The stained slices were placed in the recording chamber mounted on a microscope (Axioskop 2 FS) and perfused with oxygenated ACSF of 30˚C at a rate of 2 ml/min. The microscope was mounted on an isolation stage (Minus K Technology) on top of a stable marble table.

Slices were illuminated with a 100 W halogen-tungsten filament bulb, powered by a DC voltage source. The excitation light was filtered with a 705 ± 60 nm interference filter. Optical responses were recorded using a 464-channel photodiode array (H-469II Photodiode Array, WuTech Instruments). A 5× objective (0.25 NA Fluar, Zeiss) was used to project the slice onto the diode array. The data acquisition was controlled by a custom-made program (for details, see Wu et al., 1999). The signal from each diode was digitized at 1 kHz with a 12-bit data acquisition board (DAP 3200a/415 Microstar Laboratories). A digital image of the slice was acquired (SPOT, Imaging diagnostics) for off-line superposition of the slice morphology over the diode recording sites.

Membrane depolarization is reflected by NK3630 as a decrease in light absorption (Jin et al., 2002), which is represented in our measurements as a positive signal. The changes in light absorption [ΔA(*t*)] are proportional to the absolute light level A. To get a relevant signal with sufficient dynamic range we recorded ΔA(*t*) after high-pass filtering (>0.2 Hz) with a high-gain setting (500×) and then divided this ΔA(*t*) recorded at each diode to its absolute light level (Amax) that was recorded in a low gain setting after the transition from light-off to light-on. We assume that ΔA(*t*)/A is well approximated by ΔA(*t*)/Amax. Amax was repeatedly determined to check and correct for possible signal degradation over the time period of the recording.

*Voltage-sensitive dye data analysis.* Analysis of the data was performed using custom-made software in MATLAB (MathWorks). Diode channels recording the deep layers of the PER and LEC were selected and averaged for further analysis (Fig. 3*A*, first snapshot). Recordings of the evoked responses at a 500 µA stimulus, were averaged over at least three artifact-free, consecutively acquired realizations. Instrumentation offset, determined by the mean ΔA/Amax over a 100 ms time window before the stimulus, was subtracted from each recording. Furthermore, the recordings were filtered in space with a 2D Gaussian filter with a kernel width of one inter-diode distance (∼150 µm) and filtered in time using a running average filter with a window size of 5 ms. Positive voltage-sensitive dye signals mainly reflect the dendritic depolarization of neurons (Chemla and Chavane, 2010). We restricted the analysis in this study to the first positive reflection that was present after stimulation in all of our experiments. This positive reflection is hereafter referred to as “the response”. The undershoot following this initial response, probably as a result of a change in intrinsic properties of the slice after activity, was not further analyzed (Shoham et al., 1999).

### Whole-cell recordings in principal neurons

In total 46 principal neurons were recorded in the PER and LEC deep layers. The localization of the PER and LEC in our slice preparation was based on the mouse brain atlas (Paxinos and Franklin, 2001). Patch pipettes were pulled using micropipette puller model P-87 (Sutter Instruments) and had a resistance of 3–5 MΩ. Whole-cell recordings were performed using an intracellular solution containing the following (in mm): 131.25 K-gluconate, 8.75 KCl, 10 HEPES, 0.5 EGTA, 4 MgATP, 0.4 Na_2_GTP, pH adjusted to 7.4, 295–300 mOsm. One percent biocytin (Sigma-Aldrich) was added to the intracellular solution for *post hoc* visualization and morphologic identification of the recorded neuron. During the recordings, slices were perfused with ACSF of 30°C at a rate of 2 ml/min. Deep layer PER and LEC principal neurons were selected based on large soma size using a Scientifica SliceScope Pro 6000 (Scientifica). Whole-cell recordings were made using an AxoPatch 200B amplifier (Molecular Devices), filtered at 10 kHz, sampled at 100 kHz and digitized using a NI DAQ usb-6259 (National Instruments). Software for data-acquisition was custom made in MATLAB. All voltage signals were corrected online for a −14 mV junction potential. Principal neurons were approached with slight positive pressure on the pipette and when pressure was released the pipet-cell contact had to reach a seal of 1 GΩ before break in. Immediately after break in, the resting membrane potential was recorded in current clamp at a 0 pA holding current. Access resistance was compensated for at least 50–60% and recordings with an access resistance >20 MΩ or with >25% change during the recording were discarded.

### Decomposition of stimulus-evoked synaptic conductances in principal neurons

The evoked synaptic response in a neuron contains components that originate from excitatory and inhibitory synapses. As blocking some of these components with pharmaceuticals will affect all responses in the network, we linearly decomposed the current of principal neurons into two underlying components based on their different reversal potential. The postsynaptic cell was clamped at potentials between −90 and −50 mV, while evoking the same, voltage-independent, synaptic conductance. After subtraction of the stimulus independent background current, this results in a membrane current that contains the excitatory synaptic current and the inhibitory synaptic current:

$$I_{m}\left(t\right)=I_{exc}(t)+I_{inh}(t).$$

These currents are the result of the excitatory and the inhibitory synaptic conductances [G_exc_(*t*) and G_inh_(*t*)] and their respective driving forces: the differences between membrane voltage *V*_m_ and the reversal potentials (*E*_exc_ and *E*_inh_):

$$I_{m}\left(t\right)=G_{exc}\left(t\right)*\left(V_{m}(t)-E_{exc}\right)+G_{inh}(t)*(V_{m}(t)-E_{inh}).$$

The instantaneous relation between membrane current and membrane voltage at each moment in time can be characterized as follows:

$$I_{m}=\left(G_{exc}+G_{inh}\right)*V_{m}-(G_{exc}*E_{exc}+G_{inh}*E_{inh}).$$

The last equation is the linear *I*/*V* relation *I*_m_ = a × *V*_m_ + b, which can be calculated at each moment in time and from which the time varying conductances can now be constructed as follows:

$$G_{inh}(t)=\left(b\left(t\right)+a(t)*E_{exc}\right)/(E_{exc}-E_{inh}),$$

$$G_{exc}(t)=\left(a\left(t\right)-G_{inh(t)}\right).$$

We performed this calculation for 100 ms after the stimulus and with 0.1 ms time resolution. If there are only glutamatergic and GABA_A_ergic synapses activated and we have exact knowledge of their (time-invariant) reversal potentials (0 mV, respectively, −70 mV; Purves et al., 2001; Melzer et al., 2012), *G*_exc_ and *G*_inh_ describe the time course of the stimulus evoked synaptic conductances in the cell. The conductances induced by stimulation were averaged over three repetitions.

The instantaneous relation between the *G*_exc_ and *G*_inh_ can be examined by calculating the excitability ratio [*E*_ratio_(*t*)] at every moment in time after the stimulus:

$$E_{ratio}(t)=\left(G_{exc}+G_{m}\right)/\left(G_{inh}+G_{m}\right).$$

The membrane conductance (*G*_m_) of the cell was determined as the inverse of the passive membrane resistance recorded in voltage clamp (Table 1). The membrane conductance was added to the synaptic conductance to prevent a division by 0.

**Table 1.** Intrinsic properties of principal neurons and PV interneurons

**Property**	**Principal neurons (*n* = 30)**	**PV interneurons (*n* = 25)**
RMP, mV	−62.8 ± 0.8	−65.9 ± 0.8
Input resistance, MΩ	111 ± 8	104 ± 9
Capacitance, pF	21.4 ± 2.43	14.3 ± 1.97
Sag, mV	−2.1 ± 0.3	−0.2 ± 0.1
Time to first AP, ms	65 ± 7	24 ± 6
AP threshold, mV	−37.3 ± 0.7	−36.1 ± 0.9
Current injection, pA^a^	165 ± 17	251 ± 17
AP amplitude, mV	107.1 ± 2.0	76.7 ± 1.1
AHP amplitude, mV^b^	8.3 ± 1.0	31.1 ± 0.9
Spike half-width, ms	0.91 ± 0.03	0.49 ± 0.01
Mean firing frequency, Hz	10.3 ± 0.6	38.1 ± 2.6

RMP, Resting membrane potential; AP, action potential; AHP, afterhyperpolarization. All values are mean ± SEM. All values are measured at the current step above threshold.

a Current injection is the amplitude of the injected current evoking action potential firing.

b AHP amplitude is measured from threshold to maximal afterhyperpolarization.

### Paired whole-cell recordings of principal neurons and PV interneurons

PV-expressing interneurons in the PER and LEC network were identified using transgenic mice conditionally expressing yellow fluorescent protein (YFP) driven by the PV promotor-dependent cre-recombinase expression. YFP was excited at 470 nm using LED illumination light source (PE-100, CoolLED) and a 479 ± 40 nm emission filter (Thorlabs). Paired whole-cell recordings of one PV interneuron and one principal neuron were performed with a maximal inter-soma distance of 200 µm. The firing properties of the cells were recorded by injecting a membrane current that set the membrane voltage from −100 to −30 mV in steps of 5–10 mV.

Next, we addressed the stimulus-evoked synaptic current in voltage clamp (−70 mV) and action potential firing in current clamp in response to AiP or LA stimulation in both principal neuron and PV interneuron. The maximum stimulus intensity was 793 ± 64 µA for AiP stimulation and 967 ± 33 µA for LA stimulation, we adjusted the stimulation intensity based on the response of the principal neuron.

### Data analysis of the whole-cell recordings

Response detection was performed using MATLAB. The response was detected when the signal exceeded eight times the baseline standard deviation, within 30 ms after the stimulus was applied. If a response was detected the latency and the peak of the response were determined. The latency was determined as the time between the point where the stimulus was applied and the response was detected. The peak of the response was characterized as the maximum amplitude after the onset latency. The peak and peak time of the action potentials were determined using MATLAB (*peakdet* function; Borges, 2015), to address the presence and rate of action potential firing.

### Fitting the DUO responses to analyze summation of synaptic responses

To estimate the summation of responses when the AiP and LA were simultaneously stimulated (in this paper referred to as DUO stimulation), we took the arithmetic sum of the individual AiP and LA response and fitted this summed response onto the DUO response: DUO = α × (AiP + LA). The responses were weighted by an exponential curve, with a time constant of 20 ms, to ensure that the weight of the fit was stronger in the first 40 ms of the response. The fit was performed using a linear regression model in MATLAB (*lsqnonneg* function). This fit revealed a scaling factor α, indicating how the arithmetically summed AiP and LA response had to be scaled to configure the DUO response. If this scaling is >1, the synaptic responses after DUO stimulation summated super-linearly. If the scaling factor is 1, the synaptic responses after DUO stimulation summated linearly and if the scaling factor is smaller than 1, the synaptic responses after DUO stimulation summated sub-linearly.

### Statistics

All values are reported as mean and SEM. Statistical analysis was performed using MATLAB or Prism 6 (GraphPad Software). Unless otherwise mentioned, pairwise comparisons were made using Student's *t* test. *P* < 0.05 was assumed to reject the null hypothesis. The Pearson’s coefficient test was used to characterize the skewness of distributions.

## Results

### Principal neurons in the PER-LEC network receive synaptic input from the AiP as well as the LA

To examine whether principal neurons are recruited by the AiP or LA, we electrically stimulated the superficial layers of the AiP and the LA in horizontal mouse brain slices (Fig. 1*A*) while recording deep layer PER-LEC principal neurons. Only neurons responding to both individual AiP and LA stimulation were included for further analysis (31/46 neurons). The stimulus intensity evoking the maximum synaptic response was determined by stimulating at increasing stimulus intensities (Fig. 1*B*,*C*) and used for additional experiments. The intensity evoking the maximum response was lower for AiP stimulation (727 ± 40 µA) compared with LA stimulation (930 ± 61 µA, *p* = 0.004).

The 31 principal neurons in the PER-LEC deep layer principal neurons that received synaptic input on stimulation of either the AiP or LA had an average resting membrane potential of −62.8 ± 0.8 mV. Current injections at increasing intensities revealed the spike threshold of the recorded principal neurons, resulting in an average spike threshold of −37.3 ± 0.7 mV (Table 1; Fig. 1*D*, inset). Individual AiP and LA stimulation at maximum stimulus intensity evoked postsynaptic potentials (PSPs) in principal neurons (Fig. 1*D*,*E*). The evoked PSPs had a larger amplitude after AiP stimulation (6.4 ± 0.8 mV), compared with LA stimulus-induced PSPs (2.6 ± 0.5 mV, *p* < 0.0001; Fig. 1*D*,*E*,*G*). Although both AiP and LA inputs evoked PSPs after a single pulse stimulus, the threshold for action potential firing was not reached in the recorded deep layer principal neurons under these experimental conditions.

These results indicate that the AiP and LA are able to evoke synaptic input in the same principal neurons the PER-LEC network, although no spikes were generated under these conditions. The interplay between evoked excitation and inhibition could well play a role in the absence of principal neuron firing.

### Individual AiP and LA stimulus-evoked synaptic input consists of a small excitatory and large inhibitory conductance

**Figure 2.**
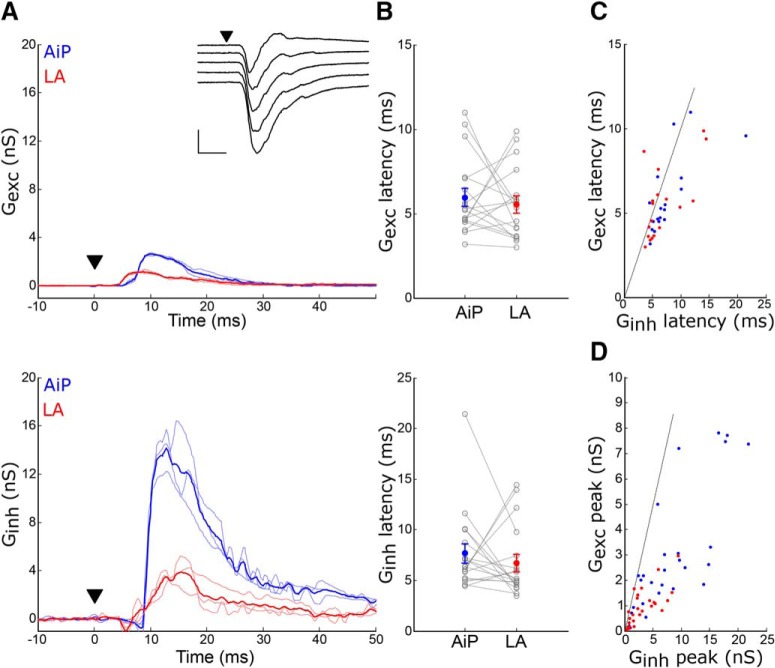
Evoked synaptic conductances in principal neurons after AiP or LA stimulation. ***A***, Example traces of the evoked *G_exc_* (top) and *G_inh_* (bottom) after AiP and LA stimulation. Inset, Evoked synaptic current at five holding potentials (−90 to −50 mV). The traces represent the average (thick line) ± SEM of three consecutive recorded responses (thin lines). Scale bars: 500 pA, 10 ms; ▼ indicates the moment the stimulus was applied. ***B***, Latency of the *G*_exc_ (top) and *G*_inh_ (bottom) evoked by AiP and LA stimulation, lines indicate the neurons recorded. ***C***, ***D***, *G*_inh_–*G*_exc_ relationship of the response latency (***C***) and peak amplitude (***D***) after AiP (blue) and LA (red) stimulation.

Next we investigated the excitatory and inhibitory input received by principal neurons in response to individual AiP and LA stimulation. The synaptic responses discussed above (Fig. 1*B*,*C*) were recorded at a membrane potential of −70 mV, which is the reversal potential for chloride, hence stimulus-evoked GABA_A_-mediated inhibition cannot be recorded at this potential. We therefore recorded the synaptic response at various holding potentials to further analyze the recruitment of the inhibitory and excitatory network by individual AiP and LA input. The evoked synaptic conductance was decomposed into the excitatory (*G*_exc_) and inhibitory conductance (*G*_inh_) in 18 principal neurons. The LA stimulation evoked *G*_exc_ latency (5.55 ± 0.50 ms) and *G*_inh_ (6.72 ± 0.82 ms) was comparable to the AiP stimulus evoked *G*_exc_ latency (5.95 ± 0.52 ms, n.s.; Fig. 2*A*,*B*, top) and *G*_inh_ (7.67 ± 0.94 ms, n.s.; Fig. 2*A*,*B*, bottom).

The excitation was evoked before inhibition as the *G*_exc_ preceded the *G*_inh_ by 1.7 ± 0.7 ms after AiP stimulation (*p* = 0.02) but the *G*_exc_ and *G*_inh_ were evoked simultaneously in response to LA stimulation (Fig. 2*C*). Furthermore, the *G*_exc_ peak (AiP: 2.94 ± 0.56 nS, LA: 0.75 ± 0.1 nS) and *G*_inh_ peak (AiP: 8.89 ± 1.93 nS, LA: 3.22 ± 0.63 nS) were determined and the peak *G*_inh_ was larger than the peak *G*_exc_ after both AiP and LA stimulation (AiP: *p* = 0.0019; LA: *p* < 0.0001; Fig. 2*D*).

These data show that deep layer PER-LEC principal neurons receive input from both AiP and LA, which consists of a small excitatory and large inhibitory conductance, with comparable timing.

### Network responses after simultaneous AiP and LA stimulation

**Figure 3.**
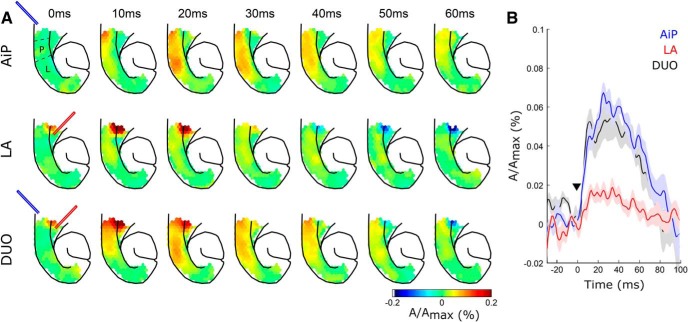
Voltage-sensitive dye (VSD) responses in the PER-EC network after AiP, LA, and DUO stimulation. ***A***, Typical example of a VSD response visualized as snapshots of the membrane voltage changes recorded at various points in time. Top row represents the VSD responses after AiP stimulation, the middle row after LA stimulation, and the bottom row after simultaneous AiP and LA stimulation (DUO). The first snapshot in the top row shows the division of the PER (P) and LEC (L). ***B***, Average (line) and SEM (shading) of the evoked VSD responses from seven experiments in PER-LEC diodes (see P and L in ***A***), evoked after AiP (blue), LA (red), or DUO (black) stimulation; ▼ indicates the moment the stimulus was applied, *n* = 7 slices.

Because deep layer principal neurons receive significant synaptic input from the AiP as well as the LA, we expect that these inputs summate in the PER-LEC network. To address this hypothesis, we stimulated the AiP and the LA simultaneously (referred to as DUO) and recorded PER-LEC network activation. We performed voltage-sensitive dye recordings of evoked neuronal activity after AiP, LA, or simultaneous AiP and LA electrical stimulation on seven acute horizontal mouse brain slices (Fig. 3*A*) to address the interaction of neuronal activity in the PER-LEC network. We found that the DUO stimulation with an electrical stimulus of 500 µA did not alter the peak amplitude of the evoked network response compared with individual AiP stimulation (AiP: 0.0741 ± 0.0038, DUO: 0.0736 ± 0.0051, n.s.; Fig. 3*A*,*B*). These data indicate that the simultaneously presented synaptic input from the AiP and LA does not evoke increased neuronal activity at the network level.

Furthermore, the amplitude of the PSPs evoked by DUO stimulation (6.2 ± 0.9 mV; Fig. 1*F*) was comparable to the AiP evoked PSP amplitude (6.4 ± 0.8 mV, n.s.; Fig. 1*D*,*F*,*G*). DUO stimulation also fails to reach the action potential firing threshold under the conditions of these experiments.

Although simultaneous stimulation of the AiP and LA does not alter total network response, the question still remains how these synaptic inputs interact and how the interplay between excitation and inhibition can be altered at the neuronal level in the PER-LEC.

### Summation of AiP and LA synaptic conductances in the PER-LEC principal neurons

**Figure 4.**
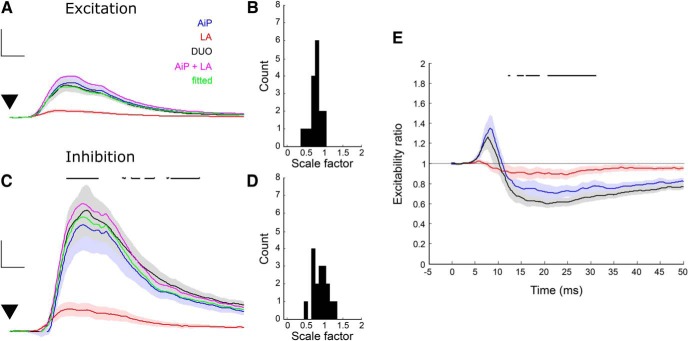
Integration of AiP and LA stimulus evoked excitatory and inhibitory responses in principal neurons. ***A***, Mean response of the evoked *G*_exc_ after AiP (blue), LA (red), and the simultaneous AiP and LA (DUO) stimulation (*n* = 18 neurons) at 100% stimulus intensity (for the results of 50% stimulus intensity, see Extended Data Fig. 4-1) and the sum of the AiP and LA *G*_exc_ (magenta, AiP + LA), with which the DUO *G*_exc_ is fitted. After fitting the DUO *G*_exc_ with the AiP+LA *G*_exc_, the AiP+LA *G*_exc_ was multiplied by its scale factor of the fit in green (fitted). The lines represent the mean and the shading SEM. Scale bars: 2 nS, 5 ms; ▼ indicates the moment the stimulus was applied. ***B***, Distribution of the scale factors calculated for the *G*_exc_ in all recorded principal neurons (in 0.1 bins). ***C***, Mean response of the evoked *G*_inh_ after AiP (blue), LA (red), and the combined AiP and LA (DUO) stimulation (*n* = 18 neurons) and the sum of the AiP and LA *G*_inh_ (magenta, AiP + LA), with which the DUO *G*_inh_ is fitted. After fitting the DUO *G*_inh_ with the AiP+LA G_inh_, the AiP+LA *G*_inh_ was multiplied by its scale factor of the fit in green (fitted). At the top, the black lines indicate the time points where the DUO evoked response was significantly (*p* < 0.05) larger than the AiP evoked response. Scale bars: 2 nS, 5 ms; ▼ indicates the moment the stimulus was applied. ***D***, Distribution of the scale factors calculated from the *G*_inh_ in all recorded principal neurons (in 0.1 bins). ***E***, Representation of the mean excitability ratio (lines, shading represents the SEM) after AiP (blue), LA (red), and DUO (black) stimulation (*n* = 18 neurons). At the top, the black lines indicate the time points where the DUO evoked response was significantly (*p* < 0.05) smaller than the AiP evoked response.

To investigate the effect of DUO stimulation on the synaptic input in principal neurons, we recorded evoked synaptic conductances and compared this with the arithmetic sum of the individual responses. We examined the evoked *G*_exc_ (Fig. 4*A*) and *G*_inh_ (Fig. 4*C*) after DUO stimulation in 18 principal neurons. To address the summation of synaptic inputs from the AiP and LA, we determined the scale factor needed to fit the arithmetic sum of the AiP and LA individual responses onto the DUO response (Fig. 4*A*,*B*). We found that the scale factor for fitting the arithmetic sum of the AiP and LA evoked *G*_exc_ onto the DUO stimulus evoked *G*_exc_ was generally <1 (mean scale factor: 0.77 ± 0.04, *Z* test with mean = 1 and SD = 0.1786: *p* < 0.0001; Fig. 4*B*), indicating that the arithmetic sum of the AiP and LA evoked excitation was larger than the response recorded at DUO stimulation. The distribution for the scale factor was slightly skewed to the left (Pearson’s skewness coefficient = −0.0527). This effect was not due to saturation of responses, because the same effect was found when responses were evoked at 50% of the maximum stimulus intensity (Extended Data Fig. 4-1), indicating that the excitatory input summated sub-linearly onto the principal neurons.

10.1523/ENEURO.0020-19.2019.f4-1 Figure 4-1 Fitting of the responses at 50% of the maximal stimulus intensity. ***A***, Typical example of the evoked G_exc_ after AiP (blue), LA (red), and the simultaneous AiP and LA (DUO), and the sum of the AiP and LA G_exc_ (magenta, AiP + LA), with which the DUO G_exc_ is fitted. After fitting the DUO *G*_exc_ with the AiP+LA *G*_exc_, the AiP+LA *G*_exc_ was multiplied by its scale factor of the fit in green (fitted). The traces represent the average (thick line) ± SEM of three consecutive recorded responses (thin lines). Scale bars: 1 nS, 5 ms. ***B***, Distribution of the scale factors for the *G*_exc_ at 50% maximal stimulus intensity (*n* = 16, 0.1 bins). ***C***, Typical example of the evoked *G*_inh_ after AiP (blue), LA (red), and the simultaneous AiP and LA (DUO), and the sum of the AiP and LA *G*_inh_ (magenta, AiP + LA), with which the DUO *G*_inh_ is fitted. After fitting the DUO *G*_inh_ with the AiP+LA *G*_inh_, the AiP+LA *G*_inh_ was multiplied by its scale factor of the fit in green (fitted). Scale bars: 5 nS, 5 ms. ***D***, Distribution of the scale factors for the *G*_inh_ at 50% maximal stimulus intensity (0.1 bins). Download Figure 4-1, TIF file.

For the DUO stimulation evoked inhibition (Fig. 4*C*,*D*) we found that in the time window around the peak *G*_inh_, the DUO response was larger than the AiP evoked response when we compared the temporal pattern of the response at multiple time points (Fig. 4*C*; *p* < 0.05). Moreover, the fit of the sum of the AiP and LA evoked responses was closer to 1 (mean scale factor 0.94 ± 0.07, *Z* test with mean = 1 and SD = 0.2785: n.s.; Fig. 4*D*), indicating that the inhibition summated linearly onto the deep layer PER-LEC principal neurons. The distribution was skewed to the right (Pearson’s skewness coefficient = 0.5254). The weights for the excitation were smaller than for the inhibition (*p* = 0.0129), once more suggesting that input is more linearly summated in the local inhibitory network.

To get an estimation of the development of the excitability over time, we calculated the excitability ratio (see Materials and Methods). Figure 4*E* shows the mean excitability ratio after AiP, LA, and DUO stimulation. The period where the ratio was >1 (*G*_exc_ > *G*_inh_) was observed in the AiP and DUO condition but the AiP and DUO response were not significantly different. In the period where the ratio was <1 (*G*_exc_ < *G*_inh_), the DUO response had a smaller excitability ratio (Fig. 4*E*; *p* < 0.05), indicating that inhibition was relatively stronger after DUO stimulation, compared with AiP-evoked inhibition. These data shows that synaptic input summates in the inhibitory response. Next, we investigated whether this evoked inhibition interacts in the interneurons in the local PER-LEC network.

### The inhibitory conductance is recruited in the local inhibitory network

**Figure 5.**
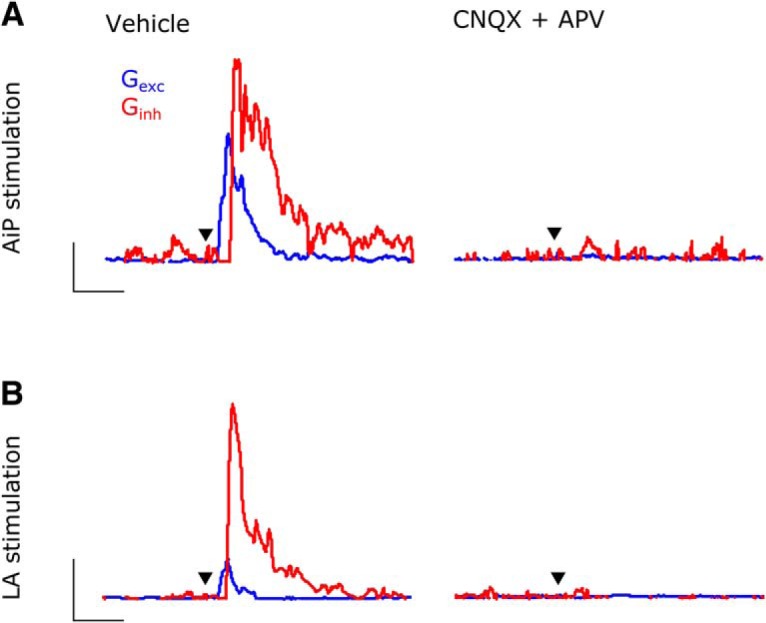
Inhibitory conductance originates from the local inhibitory network. ***A***, ***B***, Typical example of the excitatory (*G*_exc_) and inhibitory conductance (*G*_inh_) evoked by AiP (***A***) and LA (***B***) stimulation before (left) and after (right) CNQX-APV application. Scale bars: ***A***, 0.5 nS, 25 ms; ***B***: 2 nS, 25 ms; ▼ indicates the moment the stimulus was applied.

The large stimulus evoked inhibition in the recorded principal neurons by the AiP as well as the LA (Figs. 2, 4) suggests the involvement of an inhibitory network. We addressed the origin of this inhibitory response, because it can consist of a direct, long-range inhibitory connection as well as the recruitment of the local inhibitory network. To examine whether the individual AiP and LA evoked inhibitory responses were a result of local interneuron recruitment, we bath applied ACSF containing 20 µm CNQX and 10 µm APV to block the AMPA and NMDA receptor-mediated excitatory input (*n* = 4; Fig. 5). Besides blocking monosynaptic and polysynaptic excitation, this procedure also prevents polysynaptic recruitment of interneurons in the local circuitry, only allowing possible monosynaptic, long-range GABAergic projections from the AiP and LA to evoke an inhibitory response in principal neurons. It has been shown before that the inhibitory response evoked by AiP stimulation is the result of recruitment of interneurons in the local PER-LEC network (Willems et al., 2018; Fig. 5*A*). After obtaining the LA-evoked conductances in vehicle ACSF (Fig. 5*B*, left), we obtained the conductances while excitatory transmission was blocked (Fig. 5*B*, right). Both excitatory and inhibitory conductances were abolished, suggesting the absence of a direct inhibitory connection from the LA onto deep layer principal neurons in this mouse brain slice preparation. This implies that the inhibitory conductance evoked in PER-LEC deep layers by the AiP and LA input must originate from local inhibitory neurons.

### Individual AiP and LA synaptic input recruit PV interneurons in the PER-LEC network

Because the inhibition activated in principal neurons is originating from the local interneuron network, it is expected that local PV interneurons receive AiP and LA input, which they convert into action potential firing.

**Figure 6.**
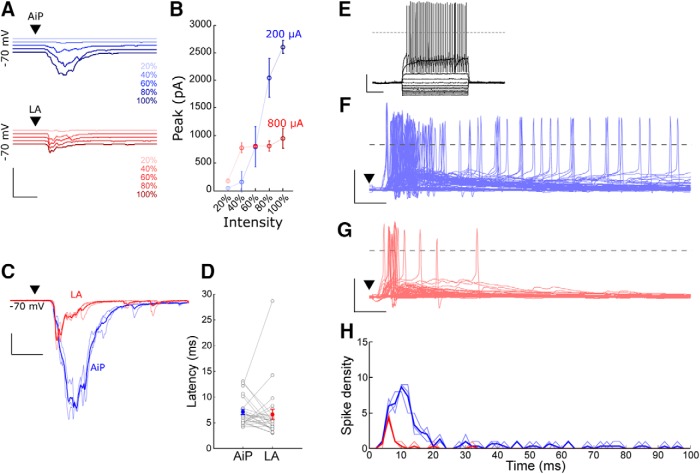
Evoked responses in PV interneurons after AiP or LA stimulation. ***A***, Typical example of evoked postsynaptic currents after AiP (blue) or LA (red) stimulation at increasing stimulus intensities. The traces represent the average of three consecutive recorded responses. Scale bars: 3000 pA, 10 ms. ***B***, Typical input/output curve of the response peak amplitude after increasing stimulus intensities in the AiP (blue) or LA (red). ***C***, Typical example of evoked postsynaptic currents after AiP (blue) or LA (red) maximal stimulation (thin lines are three consecutive recordings, thick line shows the mean). Scale bars: 500 pA, 10 ms. ***D***, The latency of the evoked synaptic current after AiP or LA stimulation (*n* = 25). ***E***, Typical example of the action potential firing of a PV interneuron evoked by injecting increasing currents. Scale bars: 25 mV, 250 ms. ***F***, ***G***, Top, raster plot of the evoked action potentials; bottom, evoked postsynaptic potentials and action potential firing after AiP (***F***) or LA (***G***) stimulation, of all recorded neurons. Scale bars: 50 mV, 10 ms. ***H***, Temporal distribution of the spike density evoked in the recorded population of interneurons (*n* = 30), after AiP (blue) or LA (red) stimulation, in 1 ms bins; ▼ indicates the moment the stimulus was applied.

We recorded the synaptic response of PV interneurons to individual AiP or LA stimulation in the PER-LEC deep layers. In total 30/30 PV interneurons responded to AiP stimulation and 25/30 PV interneurons responded to LA stimulation. Only neurons responding to both synaptic stimuli were included for further analysis (*n* = 25). The stimulus intensity evoking the maximum synaptic response was determined by stimulating at increasing stimulus intensities (Fig. 6*A*,*B*) and used for additional experiments. The intensity evoking the maximum response was lower for AiP stimulation (708 ± 51 µA) compared with LA stimulation (900 ± 33 µA, *p* = 0.00015). The latency of the LA and AiP response was comparable (AiP 7.1 ± 0.5 ms; LA 6.6 ± 1.0 ms, n.s.; Fig. 6*C*,*D*). The LA response peak amplitude (322 ± 53 pA) was smaller than after AiP stimulation (1416 ± 230 pA, *p* < 0.0001).

To examine whether the stimulus evoked synaptic input resulted in action potential firing in the recorded set of PV interneurons, we recorded 30 PV interneurons in current clamp while stimulating the individual AiP or LA. The recorded PV interneurons had a resting membrane potential of −65.9 ± 0.8 mV on average (Table 1). Current injections at increasing intensities revealed an average spike threshold of −36.1 ± 0.9 mV in the recorded PV interneurons (Table 1; Fig. 6*E*).

In total, 20 of 30 PV interneurons fired action potentials after AiP stimulation (Fig. 6*F*,*G*), whereas LA stimulation evoked firing in only 8 of 30 PV interneurons (Fig. 6*G*,*H*). In total, AiP evoked more spikes than the LA (AiP 147 spikes vs LA 22 spikes; Fig. 6*H*). The number of spikes in the recorded population of PV interneurons was largest in the first 10–20 ms after the stimulus (Fig. 6*F*–*H*).

In conclusion, both AiP and LA recruit the PER-LEC deep layer PV interneuron population. These interneurons show action potential firing on stimulation, which evokes inhibition in the local network principal neurons.

### PV Interneurons in the local PER-LEC network receive direct synaptic input after individual AiP and LA stimulation

We hypothesized that PV interneurons are directly recruited after individual AiP or LA stimulation. Therefore, we compared the timing of recruitment of PV interneurons and principal neurons in 16 recordings of principal neuron–PV interneuron pairs. In total 5 of 16 recorded pairs showed connectivity (4 principal to PV connections and 2 PV to principal connections).

**Figure 7.**
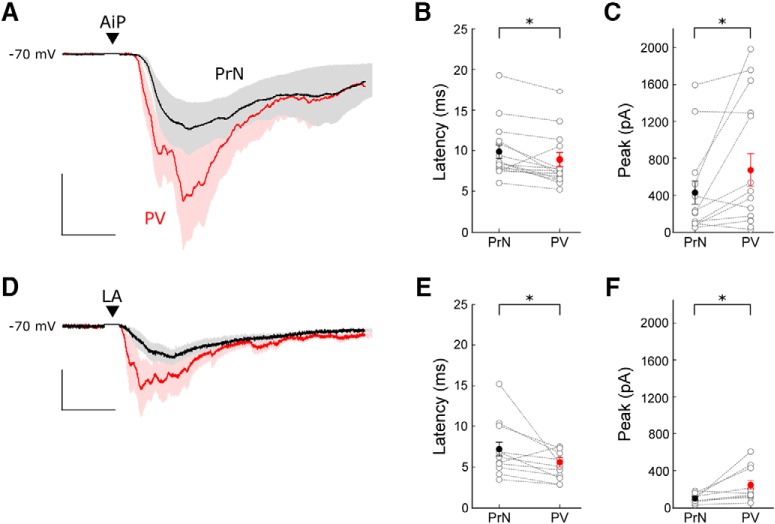
Comparison of evoked postsynaptic currents in principal neuron–PV interneuron pairs. ***A***, Typical example of the mean response of the evoked postsynaptic currents in a principal neuron (black) and a PV interneuron (red) after AiP stimulation. The traces represent the average (lines) ± SEM of three consecutive recorded responses (shaded area). Scale bars: 10 ms, 200 pA. ***B***, ***C***, Comparison of the latency (***B***) and peak amplitude (***C***) of the synaptic responses in principal neurons and PV interneurons after AiP stimulation (*n* = 16). ***D***, Typical example of the mean response of evoked postsynaptic currents in a principal neuron (black) and a PV interneuron (red) after LA stimulation. The traces represent the average (lines) ± SEM of three consecutive recorded responses (shaded area). Scale bars: 10 ms, 50 pA. ***E***, ***F***, Comparison of the latency (***E***) and peak amplitude (***F***) of the synaptic responses in principal neurons and PV interneurons after LA stimulation. Asterisks indicate the significance level (*p < 0.05). PrN, Principal neuron; ▼ indicates the moment the stimulus was applied .

The AiP and LA were stimulated at an average stimulus intensity of 794 ± 61 and 956 ± 34 µA, respectively. Consistent with earlier findings, the AiP stimulus evoked synaptic responses in PV interneurons had a smaller latency than in principal neurons (*p* = 0.0091; Fig. 7*A*,*B*; Willems et al., 2018). When the LA was stimulated, the evoked responses also arose slightly later in the principal neurons compared with the PV interneurons (Fig. 7*D*,*E*; *p* = 0.0392). The peak amplitude of the response after individual AiP and LA stimulation was larger in the PV interneurons compared with the principal neurons (Fig. 7*C*,*F*; AiP: *p* = 0.027, LA: *p* = 0.0357).

In conclusion, PV interneurons within the PER-LEC network receive synaptic input slightly earlier than principal neurons after individual AiP and LA stimulation. This, together with the absence of principal neuron firing in response to stimulation and the absence of inhibitory input when glutamatergic input is blocked, suggests that direct activation of PV interneurons results in recruitment of inhibition in a feedforward manner.

### Summation of AiP and LA responses in the PER-LEC PV interneurons

The finding that AiP and LA simultaneous stimulation leads to summation of inhibitory conductance in principal neurons (Fig. 4) together with the finding that PV interneurons are recruited after individual AiP or LA stimulation (Fig. 7), led to the hypothesis that simultaneous stimulation of the AiP and LA would summate the evoked responses in the local PV interneurons.

**Figure 8.**
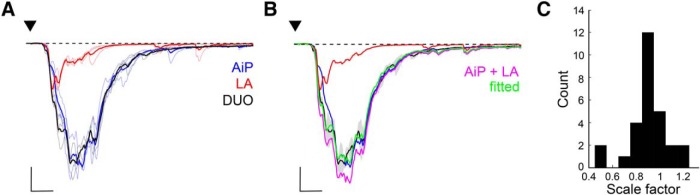
Summation of AiP and LA responses in PV interneurons. ***A***, Typical response of the evoked postsynaptic currents after AiP (blue), LA (red), and the combined AiP and LA (DUO, black) stimulation (thin lines are 3 consecutive recordings, thick line shows the mean). Scale bars: 500 pA, 5 ms. ***B***, Same typical example as in ***A***, including the sum of the AiP and LA response (magenta, AiP + LA), with which the DUO response is fitted. After fitting the DUO response with the AiP+LA response, we plotted the AiP+LA response multiplied by its scale factor of the fit in green (fitted). Scale bars: 500 pA, 5 ms. ***C***, Distribution of the scale factors calculated from the responses in all recorded PV interneurons (in 0.1 bins, *n* = 28).

To address this hypothesis, we recorded the evoked responses at a membrane potential of −70 mV after AiP, LA, and DUO stimulation in 28 PV interneurons. Figure 8*A* shows example traces of the AiP, LA, and DUO evoked responses in a single recorded PV interneuron. We fitted the DUO response with the arithmetic sum of the AiP and LA evoked responses (Fig. 8*B*) and the scaling factor was defined to fit the DUO by the arithmetically summed responses (Fig. 8*C*). We found that the simultaneously evoked synaptic responses in most of the PV interneurons were able to nearly linearly summate, with a mean scale factor of 0.91 ± 0.03 (*Z* test with mean = 1 and SD = 0.1625: *p* = 0.0035; Fig. 8*C*). The distribution was skewed to the left (Pearson’s skewness coefficient = −0.2671). Interestingly, the scaling factor was comparable to the summation we found in the inhibitory conductance evoked in principal neurons (Fig. 4).

Because the synaptic responses were recorded at the reversal potential for inhibition, these data suggest that PV interneurons accumulate excitatory synaptic input from the AiP and LA. It is therefore expected that simultaneous input from the AiP and LA changes the firing pattern of PV interneurons and hereby alter the inhibitory conductance evoked in principal neurons.

### Altered PV interneuron firing induces an inhibitory conductance shift in principal neurons after simultaneous AiP and LA stimulation

We found that principal neurons do not fire in response to DUO stimulation, which could be explained by the increase of the inhibitory input after DUO stimulation and the sublinear summation of the excitatory input in principal neurons. Additionally, the responses of the AiP and LA were linearly summated after the DUO stimulation in the PV interneuron, indicating that the interneurons would receive a larger excitatory synaptic input after DUO stimulation. Next, we addressed whether the interneuron population also showed a different firing pattern in response to DUO stimulation.

**Figure 9.**
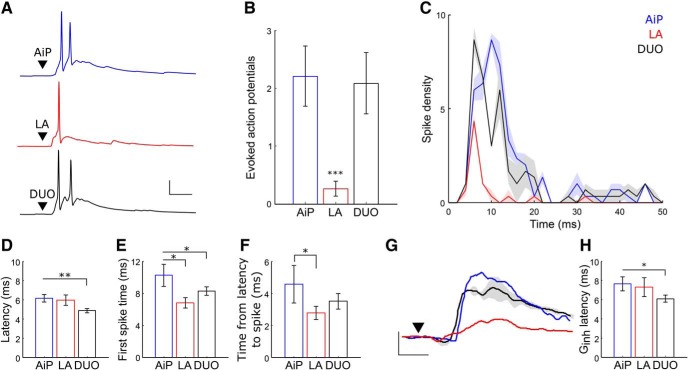
PV interneuron evoked spike patterns after AiP, LA or DUO stimulation. ***A***, Typical example of a PV interneuron firing action potentials after AiP (blue), LA (red), or DUO (black) stimulation. Scale bars: 25 mV, 10 ms; ▼ indicates the moment the stimulus was applied. ***B***, Average number of evoked action potentials after stimulation in the PV interneuron population responding to input with action potential firing in at least one stimulus paradigm. ***C***, Temporal distribution of the number of spikes evoked in PV interneurons every millisecond after AiP, LA, or DUO stimulation (thick line shows the mean, shading represents the SEM of 3 consecutive repeats). ***D***, ***F***, Comparison of the PSP latency (***D***), peak time of the first evoked spike (***E***), and the time between the PSP latency and the evoked spike (***F***) after AiP, LA, and DUO stimulation. ***G***, Typical example of the AiP, LA, and DUO evoked *G*_inh_ in a principal neuron, clearly showing the shift in the rise of the *G*_inh_ after DUO stimulation. Scale bars: 5 nS, 5 ms; ▼ indicates the moment the stimulus was applied. ***H***, The latency of the *G*_inh_ recorded in principal neurons after AiP, LA, of DUO stimulation. Asterisks indicate the significance level (*p < 0.05, **p < 0.01, ***p < 0.001).

We recorded 30 PV interneurons in current clamp and stimulated the AiP, LA, or DUO (Fig. 9*A*). In total, 19 of 30 PV interneurons showed evoked postsynaptic potentials after all three stimuli and were included for further analysis. Although LA stimulation evoked less action potentials than AiP stimulation in each neuron (AiP: 2.2 ± 0.5 action potentials, LA: 0.3 ± 0.1 action potentials, *p* = 0.0022; Fig. 9*B*), DUO stimulation evoked the same number of action potentials as AiP stimulation (DUO: 2.1 ± 0.5 action potentials; Fig. 9*B*). Considering the temporal distribution of spikes in the population of recorded neurons revealed that after DUO stimulation, the bulk of the spikes was fired slightly earlier than after AiP stimulation (Fig. 9*C*). We indeed found that the latency of the evoked postsynaptic potential was shorter for the DUO stimulus (4.9 ± 0.2 ms) compared with AiP stimulation (6.1 ± 0.4 ms, *p* = 0.0062; Fig. 9*D*). This shifted the first evoked spike forward after DUO stimulation (8.3 ± 0.5 ms), compared with the AiP stimulus (10.2 ± 1.3 ms, *p* = 0.0225; Fig. 9*E*). LA stimulation led to earlier occurrence of the first PV spike as well (6.8 ± 0.3 ms, *p* = 0.0333) compared with AiP evoked spikes, likely because of the shorter time between the latency of the response and the first spike (AiP: 4.6 ± 1.1 ms, LA 2.8 ± 0.2 ms, *p* = 0.427; Fig. 9*F*).

As the first spike of the PV interneurons shifts forward after DUO stimulation, we expect to see a forward shift of the evoked *G*_inh_ after DUO stimulation in the principal neurons. To address this, we determined the latency of the *G*_inh_ in principal neurons and found a forward shift of the *G*_inh_ latency after DUO stimulation (6.1 ± 0.4 ms) compared with AiP evoked *G*_inh_ latency (7.6 ± 0.7 ms, *p* = 0.035; Fig. 9*G*,*H*).

These data suggest that the PV interneurons are recruited faster after simultaneous stimulation of the AiP and LA, resulting in fast acting inhibition in the PER-LEC network when AiP and LA synaptic input are both activated.

## Discussion

Emotional enhancement of information processing by the amygdala is an important aspect of the memory system. How the amygdala interacts with the neuronal population in the PER-LEC of the memory system is however not yet clear. This study was designed to determine how neocortical and amygdalar synaptic inputs integrate on the neuronal level in the PER-LEC excitatory and inhibitory network. The AiP was chosen as a representative neocortical input area to the PER-LEC. The results revealed that synaptic input from the neocortical AiP and LA mainly increase the role of the inhibitory control in the PER-LEC network.

### AiP and LA have synaptic connections with the deep PER-LEC principal neurons and PV interneurons

Stimulation of the AiP or the LA activated the PER-LEC at the population level and these results are in line with previous findings. Anatomic studies showed that the AiP efferents structurally target the PER-LEC network (Mathiasen et al., 2015) and that the LA projects to both superficial and deep layers of the PER and LEC (Pitkänen et al., 2000; Sparta et al., 2014). Electrical stimulation of the AiP is known to evoke a population response in the PER-LEC network (Willems et al., 2016) and LA stimulation recruits the PER-LEC network in an *in vitro* situation as long as the inhibition in the network is slightly suppressed (Kajiwara et al., 2003; Koganezawa et al., 2008; Willems et al., 2016).

Neurons were recorded under somatic voltage-clamp conditions to quantify the excitatory and inhibitory conductances using nonlinear decomposition in response to AiP and LA stimulation. Somatic voltage clamp is certainly not perfect due to space clamp issues. Distally arriving postsynaptic currents show up in the soma with attenuated, filtered kinetics and the amplitude may be affected by an incorrect holding voltage. Because we used linear decomposition to determine conductances we could operate near resting membrane voltage, whereas errors are limited. Moreover, only neurons with a linear IV relation were considered (Poleg-Polsky and Diamond, 2016) and conductance kinetics hardly affected voltage variations. Furthermore, the *G*_inh_ and *G*_exc_ as derived from our preparation had a monophasic shape (Williams and Mitchell, 2008). AiP as well as LA stimulation evoked in principal neurons a synaptic responses that consisted of a small excitatory and a larger inhibitory conductance. The short latency excitatory component could be at least partially monosynaptic (Pitkänen et al., 2000; Pikkarainen and Pitkänen, 2001; Mathiasen et al., 2015). The excitatory component was followed by an inhibitory conductance within 1.7 ms, which is in the range of disynaptic feedforward inhibition found both *in vitro* and *in vivo* (Gabernet et al., 2005). The inhibitory input arrived slightly later at the principal neurons and it could still prevent the principal neurons from reaching their firing threshold in these particular experimental condition (Bruno, 2011; McGarry and Carter, 2016; Willems et al., 2018). The current-clamp recordings were performed at a standardized voltage level of −70 mV, to keep the PSPs comparable between neurons. Although the deep layer principal neurons do show a PSP, the threshold for action potential firing was never reached in these neurons under the current *in vitro* conditions. The difference in spiking behavior between principal neurons and PV interneurons may be related to their differences in morphology. Entorhinal deep layer neurons extend their dendritic tree into layer II/III of the cortex (van Haeften et al., 2003), whereas the dendritic trees of PV interneurons are more confined. Synaptic inputs at the distal dendrites of principal neurons could propagate less reliably to the soma, resulting in smaller EPSPs and eventually less action potential firing. Moreover, the holding potential was more hyperpolarized than the resting membrane potential, although this did not refrain the PV interneurons from spiking.

Nevertheless, the deep layer principal neurons do receive a substantial inhibitory input and the PV interneurons are considered the major source of inhibition in the PER-LEC network (Wouterlood et al., 1995). PV interneurons contribute largely to the inhibition evoked by AiP activity (Willems et al., 2018) and this study showed that LA stimulation alone activated the PV interneurons in the deep layers of the PER-LEC as well, evoking fast, large synaptic responses and action potential firing. The AiP and LA can activate the same population of PV interneurons, which implies that both pathways converge on the same PV interneurons in the PER-LEC network.

As AiP and LA converge onto the same principal neurons and PV interneurons, raises the question whether these inputs could interact on the same neurons in the deep layers of the PER-LEC network.

### Mechanism for interaction of AiP and LA inputs

Classical multisensory integration studies indicate that information across senses is integrated (Stein and Alex Meredith, 1993). These studies showed that responses of a neuron can change on stimulation of multiple sensory modalities: responses can either summate superlinear (more than the arithmetic sum of single inputs) or linear (equal to the arithmetic sum of single inputs) or sublinear (smaller than the arithmetic sum of single inputs; Stein et al., 2009). Our voltage-sensitive dye imaging experiments showed that simultaneous stimulation of the AiP and the LA resulted in a sublinear summation as could be expected if the synaptic inputs converge onto the same neurons. This was corroborated with the whole-cell recordings, which showed that the AiP and LA synaptic input converge onto the same principal neurons and PV interneurons in the PER-LEC deep layers. It is shown in humans that the amygdala and the neocortical areas can modulate the medial temporal lobe activity (including the PER and LEC) and therefore enhance memory performance of emotional stimuli (Dolcos et al., 2004). To unravel the underlying mechanism of this increased performance, the current study investigated the interaction of AiP and LA synaptic responses in the excitatory and inhibitory PER-LEC cortex neurons.

We found that the change in *G*_exc_ was summated sub-linearly, whereas the *G*_inh_ summated linearly in principal neurons. Accordingly, the synaptic input in PV interneurons summated linearly as well, although the output of the PV interneurons, assessed by the number of spikes, did hardly change. The first evoked spike in the PV interneurons shifted to an earlier point in time after simultaneous stimulation of AiP and LA, as a larger PSP reaches the threshold earlier.

In this discussion, we consider possible mechanisms that could explain the differences of the integrative phenomenon between principal neurons and PV interneurons. The linear summation as seen for the inhibitory conductances is what one would expect if the involved conductances are located at equivalent locations along the dendritic arborization or directly on the soma and the circuit activated by both stimuli does not recruit additional conductances on the neuron. For large conductances, the resulting EPSPs will summate sub-linearly due to the reduction in EPSP driving force with large depolarization. Moreover, the membrane resistance fluctuates in response to synaptic input (Gulledge et al., 2005; Tran-Van-Minh et al., 2015; Spruston et al., 2016). This may explain the sublinear summation in the excitatory conductances, as the simultaneously activated excitatory synapses that are positioned closely together, can reduce the driving force.

The sublinear summation of *G*_exc_ activated by AiP and LA stimulation in the most likely involves processes that interfere with the neuron at the network level, such as feedforward inhibition (Gulledge et al., 2005; Spruston et al., 2016). PV interneurons are known for their clustered somatic and axo-axonic projection patterns (Wouterlood et al., 1995). The observation that *G*_exc_ summated sub-linearly at 50% as well as 100% of the maximum stimulus intensity excludes an explanation that depends on saturation of responses in the network. We suggest that either inhibitory axo-axonic or presynaptic inhibition is involved. Three sources of inhibition could account for this fast inhibitory control of the simultaneous synaptic input: (1) direct inhibitory projections from the amygdala to the PER-LEC region (McDonald and Zaric, 2015), (2) direct inhibitory projections from the AiP (Pinto et al., 2006), and (3) fast recruitment of PV interneurons in the local PER-LEC network. Although direct inhibitory projections from neocortical areas and the amygdala have been shown, it is unlikely that we stimulated these inhibitory projections in our slice preparation, because complete blockade of glutamatergic transmission abolished the inhibitory responses after AiP (Willems et al., 2018) and LA stimulation. This might indicate a possible role for the PV interneurons as an important source of inhibition.

The output of the neurons was also considered in the response to simultaneous stimulation. The principal neurons could not be induced to fire and the evoked PSP was not increased compared with single AiP stimulation. Tracing studies *in vivo* and in slices showed that the AiP efferents structurally target the PER-LEC network (Mathiasen et al., 2015; Willems et al., 2016) and that the LA projects to both superficial and deep layers of the PER and LEC (Pitkänen et al., 2000; Sparta et al., 2014; Willems et al., 2016). A possible reason could be that the axonal connections between the stimulation location and the PER-LEC are cut in the brain slice, but the connectivity was (partly) functional in slices, as the voltage-sensitive dye experiments and the individual neurons do respond to AiP and LA stimulation. Moreover, comparable levels of depolarization are reported in brain slices of rats (De Villers-Sidani et al., 2004), guinea pigs (Martina et al., 2001), and gerbils (Kotak et al., 2015). We observed a larger response to AiP stimulation than to LA stimulation. This is in agreement with *in vivo* studies, which showed that the neocortex produced synaptic activation in 39% and the LA in only 25% of perirhinal cells (Pelletier and Paré, 2002; Pelletier et al., 2004, 2005). Slicing could have an effect on AiP and LA input to the PER-LEC neurons, but the observed difference in strength is in agreement with the *in vivo* situation. In the *in vivo* brain, neurons constantly receive synaptic input from a number of cortical and subcortical brain areas, resulting in the so-called high conductance state (Destexhe et al., 2003) and neurons operate much closer to threshold than in our slice. It is therefore likely that neurons are more excitable *in vivo* than *in vitro*.

The PV interneurons on the other hand, spiked in response to synaptic input and showed a unique forward shift in time of the first action potential in response to simultaneous AiP and LA input, which could induce a faster feedforward inhibition. This early inhibition shortens the temporal window in which excitatory input of the AiP and LA can coincide to result in enhancement of the EPSP. This effect could be beneficial for synchronizing activity along the PER-LEC axis when the LA is active (Pouille and Scanziani, 2001). This fast feedforward inhibition, which is homogeneously recruited by a population of interneurons, can set the threshold for firing in principal neurons and therefore select only small neuronal populations to be involved in the neuronal processing (Shadlen and Newsome, 1998). Furthermore, it is known that interneurons can provide presynaptic inhibition of excitatory synapses in olfactory sensory neurons (McGann, 2013), leading to decrease in excitatory responses recorded postsynaptically. However, whether presynaptic inhibition plays a role in the decrease of excitatory conductance after simultaneous stimulation of the AiP and LA in PER-LEC principal neurons still remains to be revealed.

In conclusion, simultaneous input from the AiP and the LA onto the deep layer neurons advanced the timing of the first spike of PV interneurons, resulting in a forward shift of inhibitory conductance evoked in principal neurons. This feature of the response interaction from the LA and AiP could therefore promote the efficacy of coincidence detection in the PER-LEC deep layer network (Pouille and Scanziani, 2001; Hu and Vervaeke, 2018).

### Functional implications

The GABAergic system in the PER-LEC deep layers is described to function as a gate, coordinating and selecting inputs from different modalities and controlling the response of principal neurons (de Curtis and Paré, 2004; Willems et al., 2018). The LA, a brain region involved in emotional memory processing (Dolcos et al., 2004), is hypothesized to facilitate information processing in the PER-LEC network (Koganezawa et al., 2008). In contrast, this study showed that the LA, while simultaneously stimulated with the AiP, did not increase the excitability of principal neurons in the PER-LEC network, but regulates the inhibitory interneuron population by shifting firing of PV interneurons forward in time. This fast recruitment of feedforward inhibition possibly creates a narrow temporal window for gating AiP activity. These results are in line with to the role of amygdala activity in gating prefrontal cortex activity for emotional behavior, by recruiting strong feedforward inhibition in the local prefrontal cortex network (McGarry and Carter, 2016). Additionally it has been shown in the hippocampus that when incoming inputs fire at low frequencies, they sum sub-linearly due to the recruitment of feedforward inhibition, whereas high frequencies sum super-linearly (Milstein et al., 2015). It is possible that the AiP and LA can cooperate the same way with the PER-LEC network, forming a high-pass filter for synaptic activity processing. Especially PV interneurons are known to be involved in shaping oscillatory activity in cortical networks, allowing signal transmission through the network (Sohal et al., 2009). The LA could possibly affect the oscillatory activity of the PER-LEC network via the fast recruitment of feedforward inhibition, which can be important for encoding of information in the cortex (Puzerey and Galán, 2014).

These results converge to the conclusion that both principal neurons and PV interneurons in the PER-LEC deep layer network receive AiP as well as LA synaptic input. These inputs often coincide on the same neurons, allowing them to integrate at the single neuron level. As a result, the feedforward inhibition recruited by a single AiP input shifts forward when the LA is active simultaneously, which likely creates a narrow time window to synchronize activity traveling through the PER-LEC network. These findings indicate a significant role for the inhibitory network in regulating integration of emotion and information for processing in the PER-LEC deep layer network.

## Synthesis

Reviewing Editor: Christian Hansel, University of Chicago Decisions are customarily a result of the Reviewing Editor and the peer reviewers coming together and discussing their recommendations until a consensus is reached. When revisions are invited, a fact-based synthesis statement explaining their decision and outlining what is needed to prepare a revision will be listed below. The following reviewer(s) agreed to reveal their identity: Vikaas Sohal, Andreas Frick.

The basic findings are that pyramidal neurons and parvalbumin interneurons in the perirhinal and lateral entorhinal cortex receive convergent input from the amygdala and insula; both inputs elicit feedforward inhibition in pyramidal neurons that is stronger than direct excitation; the main effect of simultaneous input seems to be to shift the spiking of PV interneurons, and feedforward inhibition of pyramidal neurons, forward in time. While not entirely unexpected, these are interesting and somewhat novel observations. Still, novelty is limited. Firstly, the main finding has already been shown in previous publications, namely that PV interneurons gate the information flow from the neocortex into the perirhinal cortex (e.g. Willems et al., 2018). Thus, the only true novelty in this study is the potential role that the input from the amygdala might play in this scenario, and here the authors show that amygdalar input even enhances the inhibitory brake. However, the authors have not convincingly shown that AiP input elicits significant excitatory drive in the PER under any circumstances, questioning either the question/experimental system or the experimental conditions of the study. In addition, the authors use somatic voltage clamp recordings to ‘clamp’ synaptic inputs - which technically is not possible and requires discussion as a caveat.

Detailed comments:

1. In this kind of preparation, you can't really directly compare the strength of two sources of input because many fibers may have been destroyed in the slicing procedure. I would recommend the authors remove direct comparisons of the strength of amygdala / insular inputs and focus instead on the relative strength of inhibition vs. excitation for each input.

2. The sublinear summation of the two excitatory inputs is entirely expected based on the strong inhibition evoked by both inputs. I don't really understand the alternative explanation described on lines 527-530. These factors might hypothetically explain why PV interneurons are more excitable or exhibit supralinear summation (which they don't), but they don't explain to me why pyramidal neurons have sublinear summation (in fact, h-current would tend to produce supralinear summation since one input would produce depolarization, leading to a closing of h-channels, leading to enhanced responses to the second input). I would revise this section. The only other obvious mechanism for sublinear summation here is if insular and amygdalar inputs arrive onto nearby portions of the same dendrite, producing reductions in driving force -- the authors could mention this.

3. It is quite curious that the first spike time seems to be lower for amygdala input alone compared to the “Duo” condition (Fig. 9E). This doesn't reach statistical significance, but maybe the authors could include the p-value for this and comment on it as it looks like it is close to significance, and it is somewhat unexpected. Does this perhaps reflect inhibition between PV interneurons? These factors are not discussed at all and perhaps it should be.

4. One important issue regards the methodology. The authors use somatic voltage-clamp recordings to measure excitatory and inhibitory synaptic conductances. A number of studies (e.g. Williams & Mitchell, 2008, Nature Neuroscience) have demonstrated that it is impossible to voltage-clamp neurons that possess dendrites, resulting in huge space-clamp related issues. Somatic voltage-clamp recordings only control the voltage of the soma, and therefore result in distorted measurements of synaptic inputs, the degree to which depends on the dendritic distance from the soma where the synaptic input has occurred. The authors should discuss the pitfalls of their approach and how it could affect their findings.

5. Stimulation of AiP and LA input - either alone or in combination - never reaches the threshold for AP firing in PER principal neurons. In fact, the maximum depolarization is rather small (6-7 mV) and thus far from reaching the AP firing threshold (25 mV more depolarized than the resting membrane potential). This raises several questions: Under what conditions does this input evoke firing? Is the small depolarization induced by AiP stimulation a consequence of methodological issues or suggestive of a minor role that these inputs play in driving the PER? For example, have the authors verified that the majority of AiP projections is preserved in this slice preparation? (e.g. via viral labeling). It is difficult to discuss gating of information if this information does not seem particularly relevant or other methodological issues prevent its proper investigation.

6. Related to point 2, the justification for choosing to investigate input from AiP is not sufficiently given. Why AiP and not other neocortical input?

7. In the figures, it is not clear if traces are individual traces or averages of traces; this information should be provided (e.g. Fig. 5A). Also, the traces seem very pixelated - I don't know if for initial submissions the journal policy asks for low-resolution images, but if not, higher resolution images would be helpful.

8. A recent study (Willems et al., 2018) already presents many of the findings presented here. The major novelty seems to relate to the impact of the amygdala input in the gating properties of PV-interneurons. Basically, this LA input seems to enhance the inhibitory impact of controlling excitation in the perirhinal deep layer principal neurons. Again, it is not clear to me under which conditions (except in pathophysiological conditions of dysfunctional inhibition such as epilepsy) input from AiP (and LA) even induce significant excitatory drive of PER principal neurons. The authors should discuss this in more detail.

9. Data analysis of whole-cell recordings. The authors used a very long detection time window of 75 ms for stimulus evoked responses. Could the authors please comment on this?

10. Voltage sensitive dye imaging. I am not convinced that this single experiment contributes much to the story. Furthermore, the stimulation intensity used for these experiments (500 uA) was different for the one used for whole-cell recordings, reducing its message. The authors should explain more the value of this experiment and the experimental conditions.

11. Figure 4A: Why is the latency of the responses to AiP and LA stimulation, respectively, so different in this figure (~3-4 ms)?

12. Also, the authors should indicate the resting membrane potential of the recorded neurons in the figures; e.g. Fig. 7A and 7B. Both neurons seem very depolarized and close to AP firing threshold, in particular the interneuron shown in panel B.

13. Figure 7C-H. Are the data shown from synaptically connected pairs of neurons as in A and B? If yes, which pairs are shown (P>I or I>P)? If not, what is the point of showing synaptically connected pairs in A and B?

14. The language can be improved in certain parts of the manuscript, e.g. “The period where the ratio was above 1 (Gexc > Ginh), the AiP and DUO response were comparable.”

15. Some typos, e.g. ‘PV interneurons in the local PER-LEC network are receive direct synaptic input after individual AiP and LA stimulation’.
