# Supplementary figures and images for: Interaction of Cortical and Amygdalar Synaptic Input Modulates the Window of Opportunity for Information Processing in the Rhinal Cortices
Source: eNeuro. 2019 Aug 23;6(4):ENEURO.0020-19.2019. doi: 10.1523/ENEURO.0020-19.2019 (PMC6712206; doi:10.1523/ENEURO.0020-19.2019)

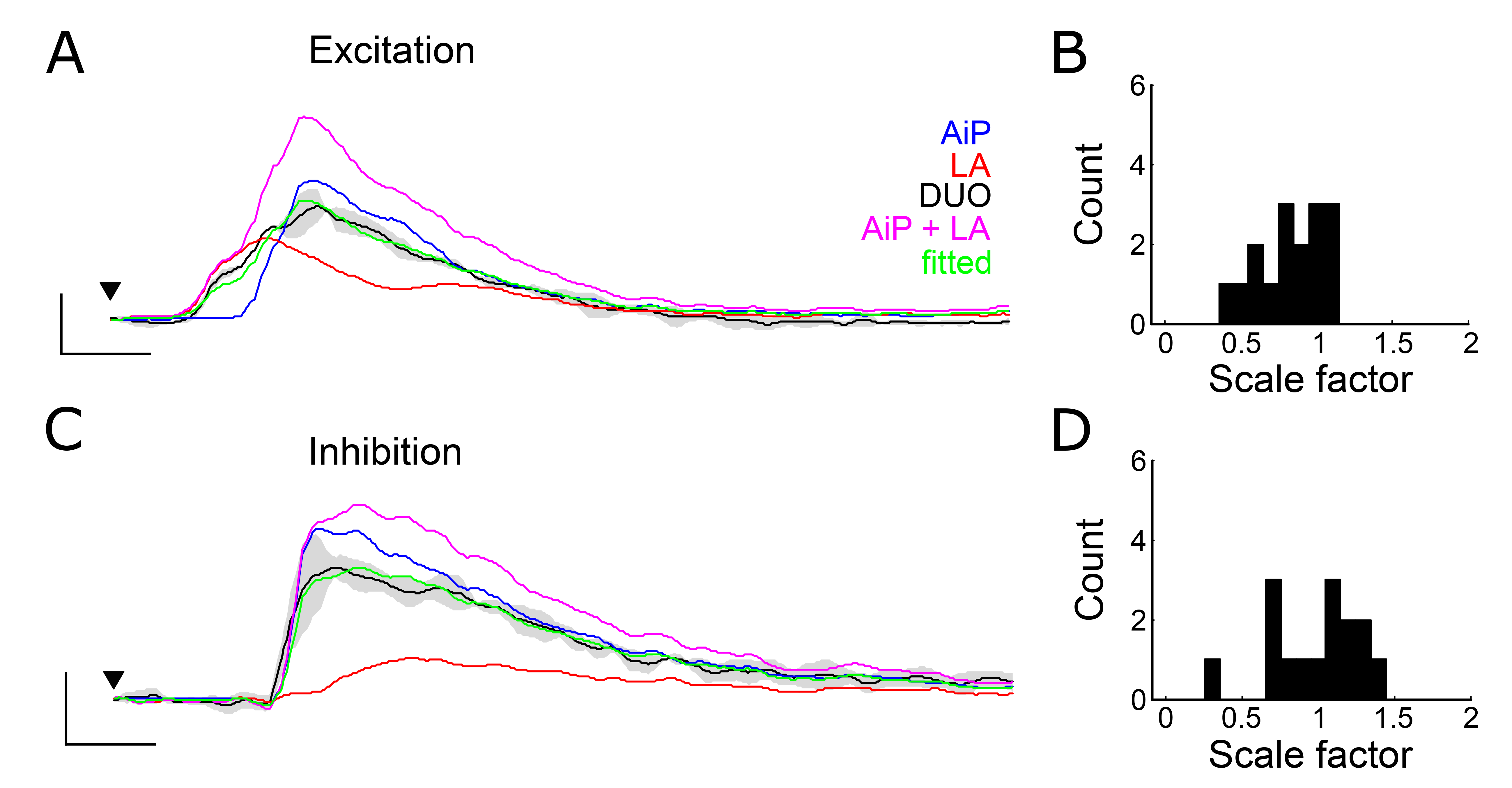

Supplement: Figure 4-1 — Fitting of the responses at 50% of the maximal stimulus intensity. A, Typical example of the evoked Gexc after AiP (blue), LA (red), and the simultaneous AiP and LA (DUO), and the sum of the AiP and LA Gexc (magenta, AiP + LA), with which the DUO Gexc is fitted. After fitting the DUO Gexc with the AiP+LA Gexc, the AiP+LA Gexc was multiplied by its scale factor of the fit in green (fitted). The traces represent the average (thick line) ± SEM of three consecutive recorded responses (thin lines). Scale bars: 1 nS, 5 ms. B, Distribution of the scale factors for the Gexc at 50% maximal stimulus intensity (n = 16, 0.1 bins). C, Typical example of the evoked Ginh after AiP (blue), LA (red), and the simultaneous AiP and LA (DUO), and the sum of the AiP and LA Ginh (magenta, AiP + LA), with which the DUO Ginh is fitted. After fitting the DUO Ginh with the AiP+LA Ginh, the AiP+LA Ginh was multiplied by its scale factor of the fit in green (fitted). Scale bars: 5 nS, 5 ms. D, Distribution of the scale factors for the Ginh at 50% maximal stimulus intensity (0.1 bins). Download Figure 4-1, TIF file. [file sup_enu-eN-NWR-0020-19-s02.tif]
